# 3-Bromopyruvate sensitizes human breast cancer cells to TRAIL-induced apoptosis via the phosphorylated AMPK-mediated upregulation of DR5

**DOI:** 10.3892/or.2022.8264

**Published:** 2022-01-17

**Authors:** Yuzhong Chen, Li Wei, Xiaojing Zhang, Xianfu Liu, Yansong Chen, Song Zhang, Lanzhu Zhou, Qixiang Li, Qiong Pan, Surong Zhao, Hao Liu

Oncol Rep 40: 2435-2444, 2018; DOI: 10.3892/or.2018.6644

Following the publication of this article, an interested reader drew to the authors attention that the western blotting data shown in Fig. 3 on. p. 2439 contained apparent anomalies; first, the protein bands shown to represent the CHOP and p-AMPK experiments in Fig. 3A were strikingly similar. Secondly, the same data bands were inadvertently included in the figure to represent the GRP78 and Bax experiments for the MCF-7 group. The authors have re-examined their original data and realized that this figure was assembled incorrectly (the CHOP and GRP78 data were inadvertently duplicated in the figure).

The corrected version of Fig. 3, showing the correct data for the p-AMPK and Bax experiments for the MCF-7 group in Fig. 3A, is shown on the next page. The authors sincerely apologize for the error that was introduced during the preparation of this figure, thank the Editor of *Oncology Reports* for granting them the opportunity to publish a Corrigendum, and are grateful to the reader for alerting them to this issue. The authors also regret any inconvenience that this mistake may have caused.

## Figures and Tables

**Figure 3.**
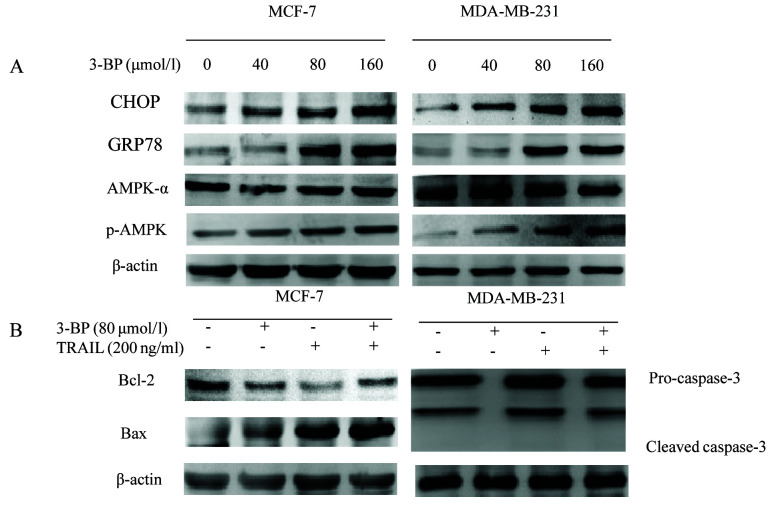
3-BP induces AMPK phosphorylation and induces cell death. (A) The levels of CHOP, GRP78, AMPK-α and p-AMPK were detected in breast cancer MCF-7 and MDA-MB-231 cells treated with 3-BP (0, 40, 80 and 160 µmol/l) for 24 h via western blot analysis. (B) The expression levels of Bax in MCF-7 cells and caspase-3 protein in MDA-MB-231 cells were detected via western blotting in cells treated with 80 µmol/l 3-BP and 200 ng/ml TRAIL. 3-BP, 3-bromopyruvate.

